# Effect of an Educational Intervention on Lifestyle Modification of Patients With Hypertension at Bishoftu General Hospital, Ethiopia, 2021

**DOI:** 10.5888/pcd20.220235

**Published:** 2023-03-30

**Authors:** Daniel Bekele Debela, Bokona Dhaba, Gemechu Shumi, Abdulnasir Abagero, Gemechu Gudina, Yadeta Ayana, Adamu Addissie, Wakgari Deressa, Angelo Scuteri

**Affiliations:** 1 Oromia Regional Health Bureau, Addis Ababa, Ethiopia; 2 Department of Preventive Medicine, School of Public Health, Addis Ababa University, Addis Ababa, Ethiopia; 3 Department of Medical Sciences and Public Health, University of Cagliari, Cagliari, Italy

## Abstract

A pilot interventional quasi-experimental study without a comparison group was conducted to evaluate the effect of a 3-month educational intervention on clinical measurement changes among 50 patients with hypertension at the Bishoftu General Hospital in Oromia Region, Ethiopia. We measured blood pressure, weight, and total cholesterol at baseline and within a week of postintervention. We found significant decreases in systolic (−12.4 mm Hg; *P < .*001) and diastolic (−4.6 mm Hg; *P < .*001) blood pressure, total cholesterol (−34.8 mg/dl; *P < .*001), and weight (−2.6 kg; *P < .*001). The educational intervention was found to be effective in reducing risk factors for cardiovascular disease.

**Summary**

**What is known on this topic?**

Lowering and controlling blood pressure are crucial in reducing risk for cardiovascular disease. The burden of hypertension is increasing in Ethiopia, and evidence of educational and counseling interventions to lower the risk is limited.

**What is added by this report?**

A 3-month educational intervention that focused on a healthy lifestyle among patients with hypertension was effective in improving systolic and diastolic blood pressure, weight, and total cholesterol.

**What are the implications for public health practice?**

Establishing an educational and counseling program for patients with hypertension in health facilities and communities would promote a healthy lifestyle and help reduce their risk for cardiovascular disease.

## Objective

Hypertension is the leading cause of cardiovascular disease (CVD) and is also a modifiable risk factor (1). Lowering and controlling blood pressure are crucial in reducing CVD-associated risks (1). In Ethiopia in 2015, the prevalence of hypertension was estimated to be 20% to 30% (2,3). The burden of hypertension is increasing because of expanding urbanization, demographic transition, and shifting lifestyle behaviors (4). A recent study reported that 48% of people in Ethiopia with hypertension had uncontrolled hypertension (5). Despite a national strategic plan to implement policies to reduce uncontrolled hypertension and improve awareness of blood pressure control, the prevention and control of hypertension have not received due attention (6).

Education and counseling interventions have been proven effective in preventing and controlling hypertension (7,8). This type of behavioral intervention includes lifestyle modifications such as promoting physical activity, a healthy diet, smoking cessation, and adherence to medication. However, most successful interventions to date have been implemented in high-income and upper-middle-income countries (7). From the available literature, we found no results on the effect of educational interventions on lifestyle modification for patients with hypertension in Ethiopia. A better understanding of the effectiveness of interventions will provide evidence for strategic health system planning in Ethiopia. Therefore, the aim of this study was to investigate the effect of an educational intervention on clinical measurements among hypertensive patients attending the outpatient department at Bishoftu General Hospital.

## Methods

A pilot interventional quasi-experimental study design without a comparison group was conducted at the outpatient department at Bishoftu Hospital in the Oromia Region of Ethiopia from January to September 2021.

We included a convenience sample of 50 patients with hypertension at the pre-intervention assessment, which corresponded with the minimum sample size required to achieve 80% power. However, only 41 participants completed the postintervention assessments and were included in the analysis. Eligibility criteria required that patients be aged 18 to 65 years, have either systolic blood pressure (SBP) of 140 mm Hg or more or diastolic blood pressure (DBP) of 90 mm Hg or more, or be taking blood pressure medication. Pregnant women and patients with comorbidities (as determined by health professionals) were excluded.

The materials for the educational intervention sessions were adapted from the World Health Organization noncommunicable diseases model and modified according to the Ethiopian context (9). All participants attended six 3-hour sessions that occurred every other week that were facilitated by nurses and physicians and consisted of didactic lectures followed by interactive group activities and exercises. The objective was to develop skills to implement a healthy lifestyle by emphasizing the importance of medication adherence and reducing hypertension-related complications. The educational messages focused on the benefits of an improved diet (reducing salt intake, including more fruit and vegetables), quitting smoking, limiting alcohol intake, increasing physical activity, and implementing behavioral change strategies for blood pressure control.

Baseline demographic characteristics were collected via an interviewer-administered questionnaire. All other measurements, including weight, blood pressure (systolic and diastolic), and total cholesterol were taken both at baseline and within 1 week after the pilot intervention. Body weight was recorded to the nearest 0.1 kg. Blood pressure was measured 3 times with the patient sitting down and the cuff placed on the left arm. Measurements were taken within 5-minute intervals, and the average of the 3 was calculated. The fasting blood samples were analyzed at the hospital using the end point Jaffe method to measure total cholesterol.

Data normality for all continuous variables was checked and confirmed as normally distributed. Data were expressed as mean (SD) or as proportions. The paired *t* test assessed overall changes in measured outcomes; the 2-sample *t* test assessed outcomes by sex. The significance level was set at *P < .*05. Statistical analyses were performed using SPSS version 24 (IBM). Ethical clearance was obtained from the ethical review board of Oromia Regional Health Bureau and written consent was taken from all participants.

## Results

Of the 50 participants, 41 completed both the pre-and post-intervention assessments for a response rate of 82.0%. The study sample was 63% female and patients had a mean age of 47.6 years. Approximately one-third (34%) of participants had no formal education, 61% were of Oromo ethnicity, 90% were Orthodox Christians, and 73% were currently married (Table 1).

**Table 1:** Sociodemographic Characteristics of Participants, Educational Intervention Among Patients With Hypertension, Bishoftu Hospital, Oromia Region, Ethiopia, 2021^a^

Sociodemographic characteristic	Baseline (N = 50)	Postintervention (N = 41)
Sex		
Female	32 (64)	26 (63)
Male	18 (36)	15 (37)
**Mean age, y**	47.6	47.6
**Age, y**		
18–29	1 (2)	1 (2)
30–44	14 (28)	11 (27)
45–65	35 (70)	29 (71)
**Ethnicity**		
Oromo	29 (58)	25 (61)
Amhara	15 (30)	10 (24)
Tigray	4 (8)	4 (10)
Other	2 (4)	2 (5)
**Religion**		
Orthodox Christian	38 (76)	37 (90)
Protestant	11 (22)	4 (10)
Muslim	1 (2)	0
**Educational status**		
No formal education	15 (30)	14 (34)
Primary education	16 (32)	14 (34)
Secondary education	12 (24)	8 (19)
College/ University completed	7 (14)	5 (12)
**Marital status**		
Never married	2 (4)	2 (5)
Currently married	37 (74)	30 (73)
Divorced	4 (8)	3 (7)
Separated/Widowed	7 (14)	6 (15)
**Employment status**		
Government employee	9 (18)	9 (22)
Private/self-employee	9 (18)	4 (10)
Housewife	12 (24)	9 (22)
Merchant	5 (10)	5 (12)
Farmer	7 (14)	7 (17)
Unemployed	8 (16)	7 (17)

a Values are no. (%) unless otherwise indicated.

From baseline to the postintervention follow-up, mean SBP decreased 9% (from 139.5 [SD, 12.9] mm Hg to 127.1 [SD, 9.5] mm Hg) and mean DBP decreased 5% (from 86.9 [SD, 7.7] mm Hg to 82.3 [SD, 5.9] mm Hg) (*P < .*001). Mean weight of participants decreased from 67.9 (SD, 12.6) kg to 65.3 (SD, 12.5) kg (*P < .*001). Total cholesterol was reduced by 17.0%, from 205.5 (SD, 26.7) mg/dl at baseline to 170.7 (SD, 27.6) mg/dl at the 3-month follow-up (*P < .*001) (Table 2).

**Table 2:** Differences in Outcomes Measures Before and After Educational Intervention Among Patients With Hypertension (N = 41), Bishoftu Hospital, Oromia, Ethiopia, 2021

Outcome measure	Baseline, mean (SD)	3-Month postintervention, mean (SD)	Mean difference	*P* value^a^
**Total**				
Systolic blood pressure, mm Hg	139.5 (12.9)	127.1 (9.5)	−12.4	<.001
Diastolic blood pressure, mm Hg	86.9 (7.7)	82.3 (5.9)	−4.6	<.001
Weight, kg	67.9 (12.6)	65.3 (12.5)	−2.6	<.001
Total cholesterol, mg/dl	205.5 (26.7)	170.7 (27.6)	−34.8	<.001
**Females (n = 26)**				
Systolic blood pressure, mm Hg	138.5 (10.7)	124.5 (9.1)	−14.0	<.001
Diastolic blood pressure, mm Hg	86.4 (5.9)	82.3 (5.9)	−4.1	.004
Weight, kg	69.7 (14.4)	64.9 (13.8)	−4.8	.002
Total cholesterol, mg/dl	206.7 (24.7)	177.1 (25.3)	−29.6	<.001
**Males (n = 15)**				
Systolic blood pressure, mm Hg	141.4 (16.5)	131.5 (8.8)	−9.9	<.001
Diastolic blood pressure, mm Hg	87.4 (10.1)	82.4 (7.0)	−5.0	.01
Weight, kg	68.3 (9.1)	66.0 (10.1)	−2.3	<.001
Total cholesterol, mg/dl	203.3 (30.5)	160.8 (26.9)	−42.5	<.001

a *P* values determined from paired *t* tests. No significant differences were found by sex using 2-sample *t* tests.

When results were stratified by sex, there were significant reductions in mean SBP (9.6%, *P < .*001) and DBP (4.5%, *P = .*004), weight (3.2%, *P = .*002), and total cholesterol (15.4%; *P < .*001) at the postintervention follow-up among females. Similarly, males had significant reductions in the mean SBP (7.7%, *P < .*001) and DBP (6.9%, *P = .*004), weight (4.9%, *P < .*001), and total cholesterol (20.1%; *P < .*001). No significant differences between sex were identified in any of these measures (Table 2).

## Discussion

We found that a 3-month educational intervention promoting a healthy lifestyle among patients with hypertension resulted in significant reductions in SBP, DBP, weight, and total cholesterol. According to the Seventh Report of the Joint National Committee on Prevention, Detection, Evaluation, and Treatment of High Blood Pressure, health-promoting lifestyle modifications are necessary to prevent the progressive rise in blood pressure and CVD (1). The lifestyle factors our intervention focused on were similar to those in the report, including reducing salt intake, increasing consumption of fruits and vegetables, increasing physical activity, avoiding alcohol, and medication adherence. Our results were consistent with findings from previous studies in high-income and upper-middle-income countries (8) and add to the evidence on the effectiveness of interventions using education and counseling strategies in CVD prevention and control in a particular context of low-income countries.

The strengths of this study included an acceptable follow-up of 82% of the study sample and objective measures obtained via a standardized protocol and results from a hospital laboratory. We anticipated explaining observed reductions in blood pressure and cholesterol as a function of weight loss. However, no significant associations were found between weight loss and blood pressure or total cholesterol, which could be because of the small sample size. Other limitations included a lack of a comparison group, a purposefully selected sample, and a short intervention duration. Hence, we cannot rule out the possibility that the changes observed from pre-intervention to postintervention were due solely to the intervention, as the improved outcomes could be attributed to one or more variables not measured.

In conclusion, our pilot study demonstrated the beneficial effect of an educational intervention on changes in blood pressure, total cholesterol, and weight in a sample of patients with hypertension. We recommend that public health practitioners develop evidenced-based educational and counseling strategies appropriate for patients with hypertension in Ethiopia who seek treatment and control services at health facilities. However, supporting community-level educational interventions to reach a larger proportion of the high-risk population is equally important. Future interventions would benefit from including a comparison group or groups and a longer follow-up period with a larger sample size to examine whether positive changes would be sustained beyond the immediate educational intervention.
